# KAP1/TRIM28: Transcriptional Activator and/or Repressor of Viral and Cellular Programs?

**DOI:** 10.3389/fcimb.2022.834636

**Published:** 2022-02-23

**Authors:** Keyera Randolph, Usman Hyder, Iván D’Orso

**Affiliations:** Department of Microbiology, The University of Texas Southwestern Medical Center, Dallas, TX, United States

**Keywords:** KAP1, Trim28, HIV-1, transcriptional regulation, epigenetic silencing

## Abstract

Several transcriptional and epigenetic regulators have been functionally linked to the control of viral and cellular gene expression programs. One such regulator is Krüppel-associated box (KRAB)-associated protein 1 (KAP1: also named TRIM28 or TIF1β), which has been extensively studied in the past three decades. Here we offer an up-to date review of its various functions in a diversity of contexts. We first summarize the discovery of KAP1 repression of endogenous retroviruses during development. We then deliberate evidence in the literature suggesting KAP1 is both an activator and repressor of HIV-1 transcription and discuss experimental differences and limitations of previous studies. Finally, we discuss KAP1 regulation of DNA and RNA viruses, and then expand on KAP1 control of cellular responses and immune functions. While KAP1 positive and negative regulation of viral and cellular transcriptional programs is vastly documented, our mechanistic understanding remains narrow. We thus propose that precision genetic tools to reveal direct KAP1 functions in gene regulation will be required to not only illuminate new biology but also provide the foundation to translate the basic discoveries from the bench to the clinics.

## KAP1 Discovery and Structure-Function Update

**Figure 1:**
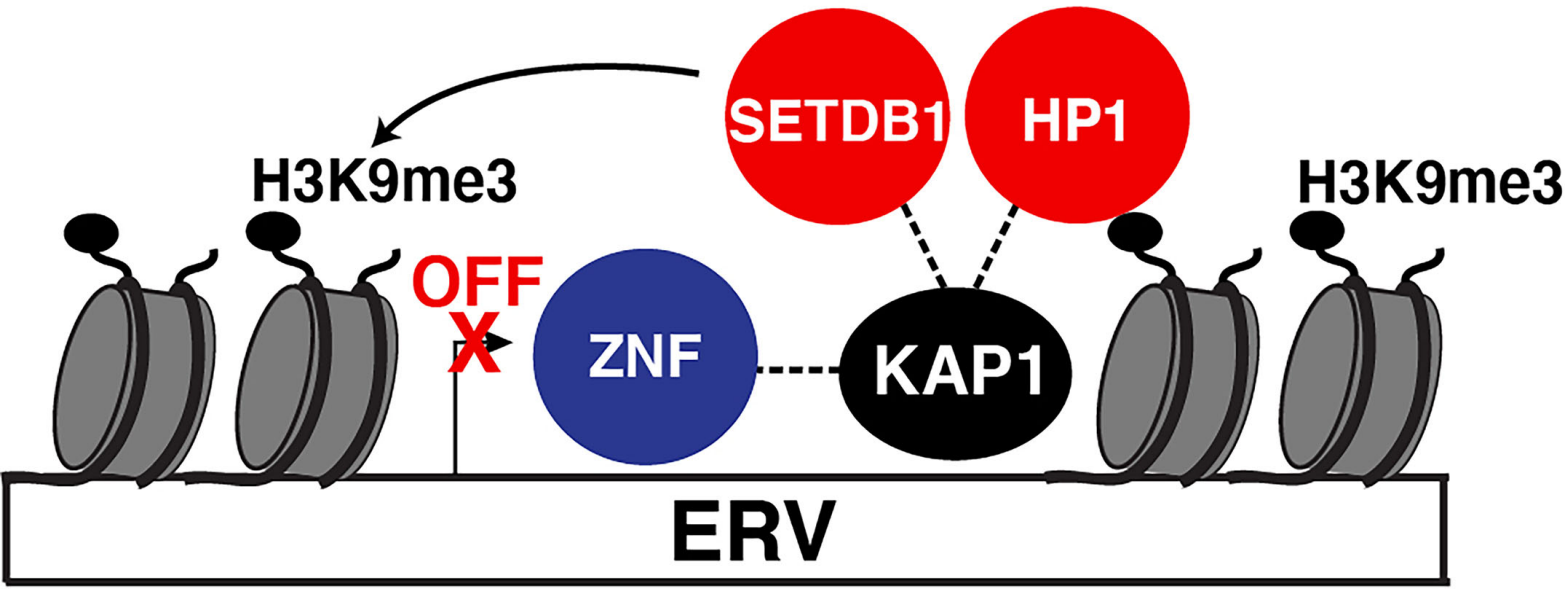
KAP1 repression of ERVs. Canonical mode of KAP1-mediated transcriptional repression of ERVs. Sequence-specific ZNF proteins recruit KAP1 which then scaffolds epigenetic silencing machinery (HP1 and SETBD1) to silence ERV expression. SETDB1 methylates H3K9 (H3K9me3) which is recognized by HP1. Both HP1 and SETDB1 co-operate to spread H3K9me3 to condense the locus and silence ERV expression.

Using proteomic screens and reporter assays, several groups in 1996 discovered KAP1 was a Krüppel-associated box (KRAB)-domain interacting protein and that fusion of KAP1 to an heterologous DNA-binding protein led to transcriptional repression of a reporter containing cognate DNA-binding sites (Friedman et al., 1996; Kim et al., 1996; Moosmann et al., 1996) in the classical artificial recruitment assay (Ptashne and Gann, 1997). Further studies identified that the primary targets of KAP1-mediated repression were endogenous retroviruses (ERVs) transcriptionally silenced in physiologically relevant cell models such as mouse embryonic stem cells (ESCs) (Rowe et al., 2010) and neural progenitor cells (Fasching et al., 2015). KAP1 deletion led to upregulation of a range of ERVs (Rowe et al., 2010). Mechanistically, KAP1 repressed ERV transcription by binding their 5’ untranslated regions through interactions with KRAB domain-containing Zinc Finger (ZNF) DNA-binding proteins, also referred to as Zinc Finger Proteins (ZFPs), which provide target DNA specificity (**Figure 1**). Upon chromatin tethering, KAP1 then scaffolds epigenetic repressive machineries, such as histone deacetylases (e.g., NuRD) (Schultz et al., 2001), Histone 3 Lysine 9 (H3K9) methyltransferases (e.g., SETDB1) (Schultz et al., 2002), and Heterochromatin-Protein 1 (HP1) proteins (Nielsen et al., 1999; Ryan et al., 1999), to promote chromatin condensation and transcriptional repression (**Figure 1**). Given the diversity of HP1 paralogs in mouse and human (HP1α, HP1β, and HP1γ) (Canzio et al., 2014) careful mechanistic interrogation is needed to define how KAP1 operates with one or all of HP1 proteins in a loci and cell-type specific manner.

Notably, ERVs are tightly regulated during development and in adulthood, as their reactivation is implicated in multiple pathologies including developmental problems, neurological disorders, and viral activation (Karlsson et al., 2001; Rowe et al., 2011; Gonzalez-Hernandez et al., 2012). As such, this repressive mechanism has been suggested to be the central means by which KAP1 regulates many of its physiological functions, including the dynamic control of both viral and cellular programs (described in detail below).

**Figure 2:**
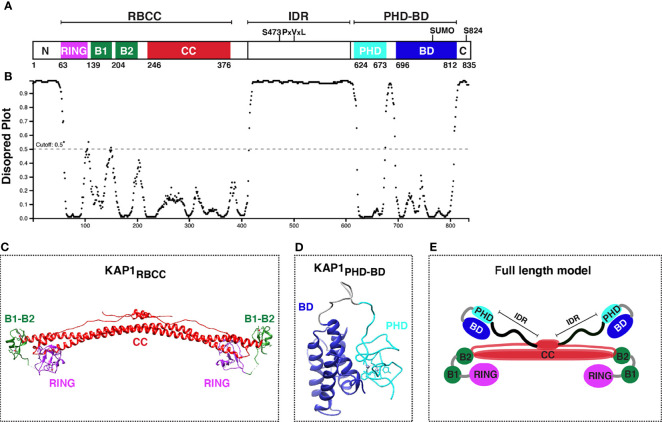
KAP1 protein domain organization and structure update. **(A)** Scheme of KAP1 protein domains with residues known to be phosphorylated (S473 and S824). PxVxL denotes the HP1-binding motif. The PHD has been reported to promote intramolecular BD SUMOylation (SUMO). **(B)** Analysis of KAP1 intrinsically disordered regions with DISOPRED3 (http://bioinf.cs.ucl.ac.uk/psipred/). **(C)** KAP1 RBCC structure, PDB 6QAJ (Stoll et al., 2019). **(D)** KAP1 PHD-BD structure, PDB 2RO1 (Zeng et al., 2008). **(E)** KAP1 homo-dimer asymmetric model adapted from Stoll et al. (Stoll et al., 2019).

KAP1 is an 88.5 kDa protein containing several well-conserved motifs (**Figure 2A**) that could engage into multivalent interactions to facilitate gene regulation. As a member of the TRIpartite Motif (TRIM) family (Nisole et al., 2005; Sardiello et al., 2008), KAP1 has a conserved N-terminal architecture consisting of a Really Interesting New Gene (RING) E3 ubiquitin ligase domain (Borden and Freemont, 1996; Doyle et al., 2010), two B-box domains involved in higher-order oligomerization (Stoll et al., 2019; Sun et al., 2019), and one antiparallel coiled-coil (CC) domain required for dimerization (Stoll et al., 2019; Fonti et al., 2019), collectively known as the RBCC domain. The RBCC domain is followed by a ~200 amino acid intrinsically disordered region (IDR) (**Figures 2A, B**) that contains an internal PxVxL motif involved in HP1-binding for gene repression (Ryan et al., 1999; Sripathy et al., 2006), and a C-terminal tandem plant homology-bromodomain (PHD-BD) cassette involved in intramolecular BD SUMOylation (Ivanov et al., 2007) and chromatin binding (Bacon et al., 2020). This RBCC–IDR–PHD-BD structure is characteristic of a subset of TRIM proteins called the Transcription Intermediary Factor 1 (TIF1) subfamily (McAvera and Crawford, 2020), which consists of KAP1 (TIF1β), TRIM24 (TIF1α), TRIM33 (TIF1γ), and TRIM66 (TIF1δ). Three TIF1 family members (KAP1, TRIM24, and TRIM33) have been reported to form hetero-dimer or -trimer protein complexes (Herquel et al., 2011; Fong et al., 2018), and KAP1 has been shown to bind TRIM24 to protect it from proteasome-dependent degradation (Fong et al., 2018). Despite hetero-complex formation and structural similarities among TIF1 family members, they have distinct interacting partners, diverse functional properties in various contexts, and appear to recognize different histone marks (Tsai et al., 2010; Agricola et al., 2011; Xi et al., 2011; Bacon et al., 2020).

The structures of several KAP1 domains have been solved by NMR and X-Ray crystallography. These include the complete RBCC (PDB 6QAJ) (Stoll et al., 2019) (**Figure 2C**) and tandem PHD-BD (PDB 2RO1) (Zeng et al., 2008) (**Figure 2D**), in addition to the minimal RING (PDB 6I9H) (Stevens et al., 2019), B-box1 (PDB 6O5K) (Sun et al., 2019), and PHD (PDB 1FP0) (Capili et al., 2001) domains. While a full-length KAP1 structure does not exist, perhaps owed to the long IDR connecting the RBCC and PHD-BD (**Figures 2A, B**), KAP1 has been biochemically and biophysically characterized as a functional asymmetric homodimer (Fonti et al., 2019; Stoll et al., 2019) (**Figure 2E**).

**Figure 3:**
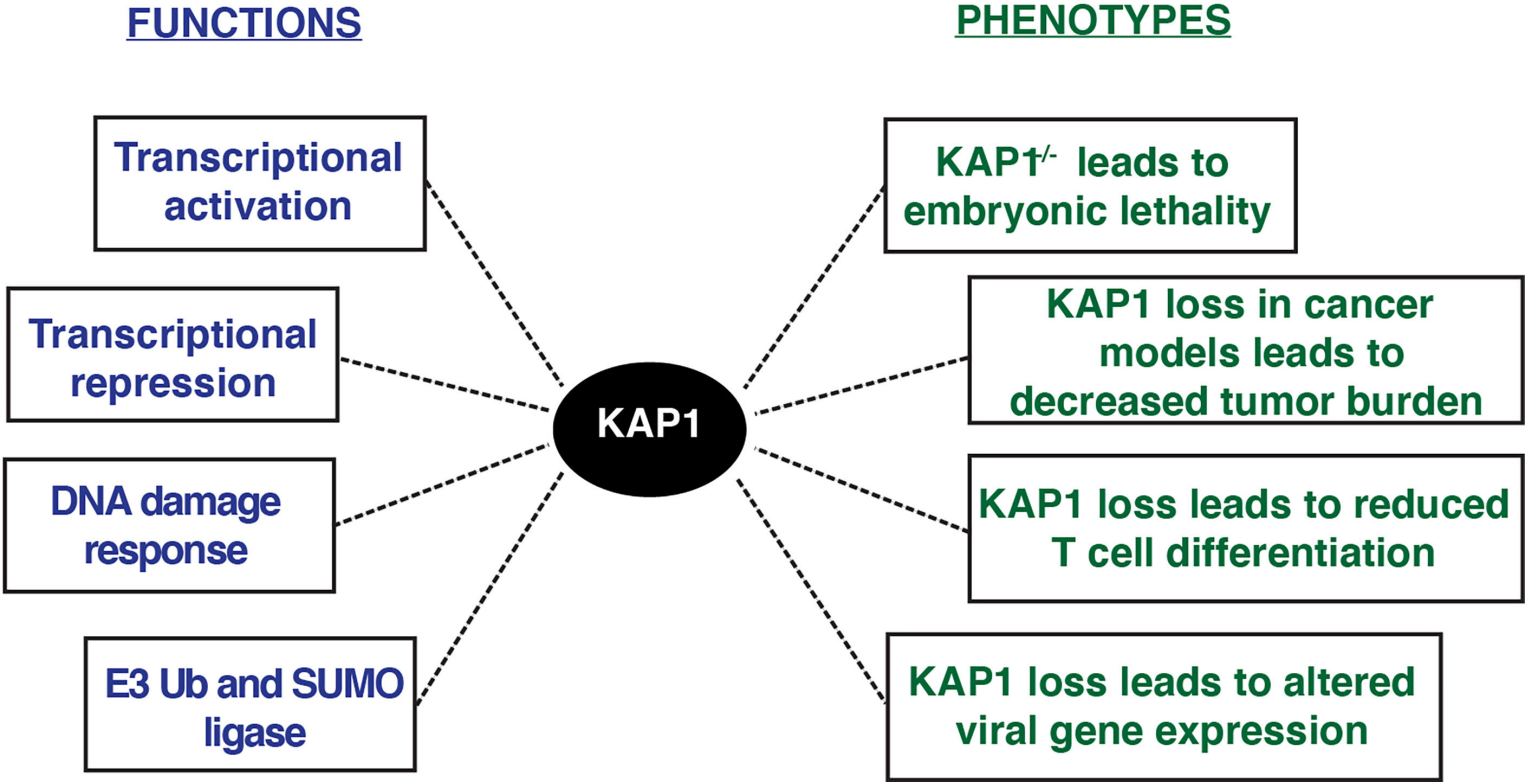
KAP1 functions and phenotypes described upon KAP1 loss in cell and mouse models.

Functionally, multiple KAP1 roles have been described in a variety of phenotypic contexts (**Figure 3**). KAP1 is ubiquitously expressed in every human tissue throughout development and adulthood (Human Protein Atlas), and KAP1 knockout mice are embryonic lethal at E8.5, highlighting its important role in early development (Cammas et al., 2000). Potentially explaining this lethality, mouse ESCs with KAP1 depletion undergo rapid differentiation suggesting that KAP1 is required for stem cell maintenance (Hu et al., 2009; Seki et al., 2010; Cheng et al., 2014). Despite these robust phenotypic observations, it remains unclear which KAP1-dependent mechanisms, either KAP1 transcriptional regulation of ERVs and/or pluripotency-associated genes, drive the phenotypes observed upon KAP1 loss in stem cell models.

KAP1 functions have also been studied in a variety of pathological contexts, including cancer (Czerwinska et al., 2017). KAP1 RING domain has E3 ubiquitin ligase activity on substrates including p53 and the AMPK tumor suppressor, highlighting potential oncogenic KAP1 roles (Wang et al., 2005; Doyle et al., 2010; Pineda et al., 2015). Further, many studies have shown KAP1 loss in various tumor models decreases cancer cell growth (Li et al., 2017a; Fong et al., 2018). Consistent with these molecular studies, KAP1 is amplified in most human cancers, and increased KAP1 expression correlates with poor patient prognosis in multiple tumor types including ovarian, lung, and glioma (Liu et al., 2013; Cui et al., 2014; Wang et al., 2016; Su et al., 2018). Additionally, KAP1 haploinsufficiency triggered bi-stable epigenetic obesity (Dalgaard et al., 2016), and KAP1 disruption elicited spermatogenesis (Herzog et al., 2011) and erythropoiesis defects (Barde et al., 2013). Altogether, KAP1 is implicated in a variety of physiological processes critical for normal homeostasis with implications in disease progression (**Figure 3**).

**Figure 4:**
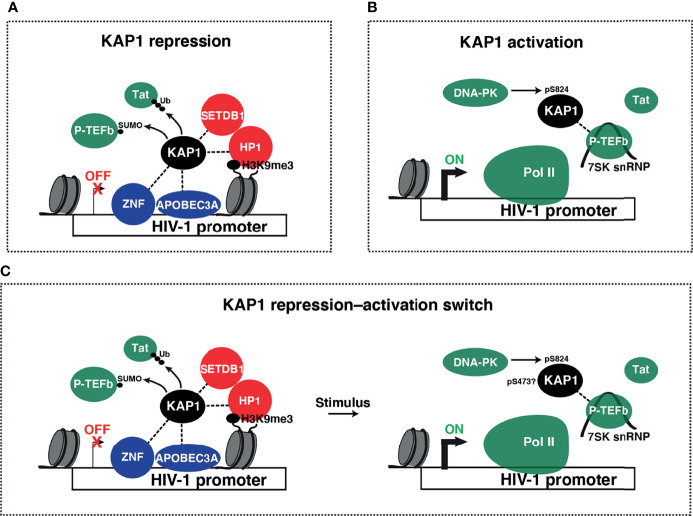
Models of KAP1 transcriptional repression and activation. **(A)** Collective ideas derived from studies reporting KAP1-mediated HIV-1 transcriptional repression. KAP1 was shown to be recruited to the HIV-1 LTR by many factors including ZNF proteins (ZBRK1, ZNF10, and ZNF304) and APOBEC3A. KAP1 then either recruits heterochromatin machinery (SETDB1 and HP1α) or negatively regulates P-TEFb (through SUMOylation) and/or Tat (through ubiquitination and proteasome-mediated degradation) to repress HIV-1 transcription in basal and stimulated conditions. **(B)** Proposed model for KAP1-mediated HIV-1 transcriptional activation. KAP1, which may be phosphorylated by DNA-PK, scaffolds P-TEFb in its inactive state (assembled into the 7SK snRNP complex) in a Tat-independent manner to regulate Pol II pause release thereby promoting HIV-1 activation. **(C)** Paradigm of KAP1-mediated transcriptional repression-to-activation switch.

Despite the longstanding transcriptional repressive function, many groups have identified KAP1 to also be a transcriptional activator of viral and immune cell programs. As KAP1 transcriptional repressive functions during development and other contexts have been reviewed elsewhere (Iyengar and Farnham, 2011), in this review we will focus on the diverse and controversial roles that KAP1 plays in the activation of viral and cellular transcriptional programs. We hope to shed light on the pleiotropic nature of KAP1 functions and probe the paradigm for KAP1 functioning as transcriptional repressor, activator, and/or repressor-activator switch (**Figures 4A–C**). Finally, given the multiple modes of KAP1 transcriptional regulation, we propose that precise genetic tools are needed in the field to properly delineate causality in the mechanisms of KAP1-mediated gene expression control.

## KAP1 Controversial Roles in HIV-1 Gene Expression and Latency Control: Activator and/or Repressor?

### KAP1 Unexpected Role in HIV-1 Transcriptional Activation

The discoveries that KAP1 silences ERVs and retroviruses (Wolf and Goff, 2007; Wolf and Goff 2008b; Wolf and Goff, 2009; Rowe et al., 2010; Rowe et al., 2013b) prompted studies to investigate if KAP1 directly represses HIV-1 proviral transcription in terminally differentiated cells. To this end, in 2016 McNamara et al. (McNamara et al., 2016) stably silenced KAP1 expression using shRNA-mediated RNAi in an immortalized CD4^+^ T cell line (Jurkat) E4 model of latency (J-Lat) bearing a single integrated provirus (Pearson et al., 2008). Surprisingly, this study revealed that chronic loss of KAP1 did not spontaneously reactivate latent HIV-1 proviruses from this model in the absence of any stimulation. Contrary to the expected repressive role, loss of KAP1 led to a partial decrease of HIV-1 gene expression in both basal conditions and in response to the pro-inflammatory cytokine TNF-α (~1.5-4–fold depending on the time point evaluated). These data suggest that in this context KAP1 is a positive regulator of HIV-1 expression or that KAP1 is needed to maintain an optimal T cell state for HIV-1 transcription. Mechanistically, biochemical and cell biological evidence in HEK293T cells supported a model whereby both endogenous and ectopically expressed KAP1 interacts with components of the transcription elongation machinery, namely the positive transcription elongation factor kinase (P-TEFb, composed by the CDK9 kinase and cyclin T1 subunit) as part of the 7SK small nuclear ribonucleoprotein (7SK snRNP) complex (**Figure 4B**). KAP1 was found to directly bind the LARP7 subunit of the 7SK snRNP but not to P-TEFb, which only co-purifies with KAP1 as part of 7SK snRNP. They then provided genetic evidence demonstrating KAP1 and 7SK snRNP co-occupy promoter-proximal regions alongside paused Pol II in the E4 cell model prior to T cell stimulation, which was then cross-validated in several other models of latency in which HIV-1 is integrated into euchromatic regions. Conversely, KAP1 and the 7SK snRNP were not detected in proviruses integrated into chromatin-dense regions or bearing core promoter mutations preventing transcription machinery occupancy, thereby indicating the requirement of transcription for KAP1 and transcription elongation complex recruitment. They then provided genetic evidence that KAP1 loss in the E4 cell model led to decreased occupancy of P-TEFb and 7SK snRNP components at the HIV-1 5’-LTR (Long Terminal Repeat) in basal conditions, suggesting KAP1 participates, directly or indirectly, in their recruitment. Consistent with the proposed model, loss of KAP1 in a cell model of latency lacking Tat (2B2D) (Pearson et al., 2008) diminished recruitment of P-TEFb and Pol II to the promoter-proximal region in response to TNF-α, without largely affecting NF-κB occupancy, indicating the transcriptional defects were a direct consequence of loss of P-TEFb recruitment. Broadening the scope of these discoveries, KAP1 loss dampened activation of P-TEFb–dependent, NF-κB activated pro-inflammatory target genes in CD4^+^ T cell lines and KAP1 and the 7SK snRNP co-occupied most promoter-proximal regions with paused Pol II in HCT116 cells, perhaps illuminating the discoveries with HIV-1 can be extended to cellular genes, both constitutive and signal-inducible. While McNamara et al. displayed a functional genetic interaction between KAP1 and P-TEFb in J-Lat cells, the authors did not validate the biochemical interaction in this cell system. Additionally, McNamara et al. quantified HIV-1 transcripts, but not viral proteins to determine if reduced transcription upon KAP1 loss led to protein production and/or viral defects. Together, these studies offered the first mechanistic link describing a role for KAP1 as transcriptional activator of HIV-1 in response to pro-inflammatory stimulation.

### KAP1 Activation of the “Host” Phase of the HIV-1 Transcriptional Program

Given that KAP1 activated HIV-1 in response to stimulation and because the HIV-1 transcriptional program has two regulatory phases (host and viral), Morton et al. investigated at what level(s) KAP1 precisely functions to activate the latent provirus (Morton et al., 2019). During the “host phase”, sequence-specific transcription factors like NF-κB bind *cis*-elements at the 5’-LTR and promote assembly of the transcription initiation machinery to activate HIV-1 transcription in response to pro-inflammatory stimulation (Shukla et al., 2020). This low-level transcription induces the synthesis of the viral-encoded Tat protein, which in the “viral phase”, activates HIV-1 transcription elongation and induces a positive feedback loop to potently activate the virus. Given this knowledge, the authors took advantage of J-Lat cell models of latency bearing single copies of integrated proviruses with wild-type Tat (E4), to monitor KAP1 contributions to the complete host-viral transcriptional circuit, or non-functional Tat (2B2D), to monitor KAP1 contributions to the host phase only. Morton et al. found that KAP1 loss through RNAi largely dampened HIV-1 expression in response to TNF-α in the Tat minus provirus signifying that KAP1 works in tandem with NF-κB to initially activate the host phase, consistent with data of the McNamara et al. study (McNamara et al., 2016). Expectedly, loss of KAP1 also decreased HIV-1 expression in the Tat containing provirus potentially attributed to diminished host phase initiation. Because the magnitude of HIV-1 expression loss in the Tat minus provirus was much larger than the drop in the Tat expressing virus, these experiments suggested Tat may operate in a KAP1-independent manner to transcriptionally activate the provirus in the viral phase. Notably, this model was cross validated with two complementary approaches. First, using minimalistic reporter assays in which Tat equally activated HIV-1 LTR-driven luciferase reporters in U2OS cells expressing or lacking KAP1. Second, by ectopically delivering Tat into J-Lat cells harboring the Tat minus provirus. To further characterize KAP1’s HIV-1 activating role, Morton et al. developed a mathematical framework to model a complete HIV-1 transcriptional program by incorporating the host phase into existing models simulating the viral phase (Weinberger et al., 2005). In this model, loss of KAP1 diminished HIV-1 RNA synthesis during the host phase, and fluctuations in KAP1 levels influenced the outcome of the host phase thereby diminishing Tat activation in the viral phase, which was experimentally validated with Jurkat HIV-1 clones that expressed KAP1 at various levels. In addition to characterizing which phase of HIV-1 transcription KAP1 regulates, this study conducted a series of experiments to further describe KAP1 as a transcriptional activator of the HIV-1 provirus. The authors used CRISPR-Cas9–mediated knockout of KAP1 in a CD4^+^ T cell primary model of latency to show that KAP1 loss decreased HIV-1 expression after Phorbol Myristate Acetate (PMA) stimulation (Protein Kinase C agonist), consistent with the RNAi studies in the J-Lat models (Morton et al., 2019). Additionally, KAP1 was also shown to mediate activation in response to latency reversing agents (LRAs) through different mechanisms such as Bryostatin (a PKC agonist) and suberoylanilide hydroxamic acid -SAHA- (a pan-histone deacetylase inhibitor). These data together imply KAP1 activates the host phase in response to strong immune modulators (TNF-α and PMA) as well as commonly used LRAs. Finally, loss of KAP1, which decreases P-TEFb recruitment to the HIV-1 proviral genome, can be rescued by artificially tethering P-TEFb using the yeast GAL4 system. This data suggests that by rewiring the HIV-1 transcriptional program to operate through promoter-bound P-TEFb, KAP1 becomes dispensable for activation, as in the viral phase. Nonetheless, because the reporter system used herein is artificial (GAL4–HIV-1 LTR), this result does not provide quantitative evidence that KAP1 scales with P-TEFb recruitment and does not rule out KAP1 could play other essential functions in the gene expression cycle.

### KAP1 Site-Specific Phosphorylation and a Possible Activating Connection

Other reports have been in line with these previous studies suggesting KAP1 has an HIV-1 activating role. In 2020, Zicari et al. have reported that the activity of the DNA damage response (DDR) DNA-dependent protein kinase (DNA-PK) is broadly required for latent HIV-1 transcription activation (Zicari et al., 2020). In this work, DNA-PK activity was found to be required (directly or indirectly) for KAP1 Ser824 phosphorylation (pS824-KAP1) (**Figure 2A**) and for recruitment of Pol II, P-TEFb, and pS824-KAP1 to the HIV-1 5’-LTR upon TNF-α stimulation (**Figure 4B**). As such, their model indirectly suggests that DNA-PK promotes the recruitment of KAP1 to the proviral genome to assist the release of paused Pol II through KAP1 site-specific phosphorylation. The involvement of DNA-PK in KAP1 phosphorylation has been previously documented (Bunch et al., 2014; Bunch et al., 2015). Bunch et al. studied the mechanisms of transcriptional elongation in stimulus-inducible genes and reported the enrichment of pS824-KAP1 and the DNA damage-dependent histone H2A variant (γH2AX) on serum-inducible genes. They also showed a role for DNA-PK in the release of paused Pol II and transcriptional activation-coupled DDR signaling on these genes, altogether suggesting transcriptional elongation requires DNA break-induced signaling involving the functional interplay between KAP1 and DNA-PK (Bunch et al., 2015). In line with these studies, another group recently showed that DNA-PK phosphorylates KAP1 at Ser824 to activate transcription of hypoxia-inducible genes by recruiting CDK9 to hypoxia response elements in a HIF1-dependent manner (Yang et al., 2022), consistent with the McNamara et al. model for HIV-1 transcriptional activation (McNamara et al., 2016). While it has not been formally tested by Zicari et al., the fact that DNA-PK inhibition elicited loss of transcription machinery (including KAP1) at the HIV-1 5’-LTR, supports the proposed KAP1 activating role (McNamara et al., 2016; Morton et al., 2019). Altogether, it will be interesting to determine the precise mechanism by which DNA-PK and KAP1 cooperate to activate latent HIV-1 proviruses in response to T cell stimulation and what are the specific roles of KAP1 site-specific phosphorylation. A model that summarizes the McNamara et al., Morton et al., and Zicari et al. studies is presented in **Figure 4B**.

### KAP1 SUMOylation of P-TEFb and Possible HIV-1 Transcriptional Repression

Unlike the three previous reports supporting KAP1 functions as an HIV-1 transcriptional activator, studies by Ma et al. allowed them to propose a model whereby KAP1 promotes HIV-1 latency maintenance in basal conditions (Ma et al., 2019). They initially performed a targeted siRNA screen to silence the expression of 182 human genes (using pools of 3 distinct siRNAs per gene) in the HeLa TZM-bl reporter cell line, a HeLa derivative expressing CD4 and CCR5 receptors and bearing an integrated HIV-1 LTR luciferase and β-galactosidase reporters. KAP1 was one of the several human genes whose silencing derepressed the HIV-1 LTR promoter (~4-fold) without any exogenous stimulation. They then validated this data by chronically silencing KAP1 expression using shRNA-mediated RNAi in J-Lat 10.6 cells (containing a full-length but replication-defective HIV-1/GFP genome), which yielded HIV-1 proviral de-repression (~1.4% to ~8.5% GFP-positive cells, ~6-fold) without any exogenous stimulation. These results contradict the results by McNamara et al. and Morton et al. in which KAP1 silencing with RNAi vectors in various cell models of latency did not trigger any spontaneous latent HIV-1 reactivation. Further, Ma et al. observed that while both SAHA and JQ1 augmented the percentage of GFP-positive cells in J-Lat 10.6 control shRNA (14.1% to 21.9% in SAHA-treated cells and 14% to 27% in JQ1 treated cells over mock treated cells), these LRAs only induced the percentage of GFP-positive cells in KAP1 shRNA by ~2.6-to-3.2-fold (8.5% to 21.9%-27.4%), respectively. Consistent with the studies by McNamara et al., Morton et al., and Zicari et al. KAP1 appeared to occupy the HIV-1 proviral genome (McNamara et al., 2016; Morton et al., 2019; Zicari et al., 2020). In agreement with the latent HIV-1 de-repression phenotype observed in the HeLa TZM-bl model, ChIP assays revealed repressive epigenetic marks (H3K9me2, H3K9me3, and H3K27me3) decreased ~2-fold and activating epigenetic marks (H3K4me3 and H3K9ac) increased ~2-fold upon KAP1 siRNA-mediated silencing relative to control siRNA. Given KAP1 has E3 SUMO ligase activity (Ivanov et al., 2007), Ma et al. then interrogated which protein domains link KAP1-mediated SUMOylation with HIV-1 reporter silencing in the HeLa TZM-bl cell model. By eliminating the expression of endogenous KAP1 with siRNA and complementing with wild-type and deletion constructs, their data suggested that none of the fragments were able to restrict HIV-1 silencing to wild-type levels, but that deletion of some domains (e.g., RING) (**Figure 2A**) compromised KAP1-mediated HIV-1 silencing, potentially suggesting that the RING domain is required for KAP1-regulated reporter silencing. Given these data, the authors then predicted the RING domain to be required for SUMOylation of HIV-1 activating factors to repress their function. To identify these factors, they then ectopically expressed KAP1, the SUMO E2 ligase UBC9, and SUMO1/2/4 point mutants in HeLa cells to identify SUMO-acceptor Lysines by tandem mass spectrometry. While this approach revealed ~1,300 statistically significant SUMOylated proteins, they focused their studies on one substrate (CDK9). Ectopically expressed KAP1 preferably SUMOylated CDK9 with SUMO4 compared to SUMO1 and SUMO2, consistent with the idea that SUMO4 was found in the siRNA targeted screen alongside KAP1. Additionally, the KAP1 RING domain was required for CDK9 SUMOylation *in vitro* (at Lysines 44, 56 and 68), which weakened the interaction between CDK9 and its cyclin partner (CyclinT1) thereby inhibiting P-TEFb kinase activity. The authors finally extended their data in cells to show that KAP1 silencing from aviremic patient resting CD4^+^ T cells reactivated latent HIV-1. Taken together, Ma et al. interpreted their data by proposing a model whereby KAP1 inhibits HIV-1 gene expression by SUMOylating CDK9 and that loss of KAP1 de-represses CDK9 for HIV-1 proviral activation. This model has countered previous reports suggesting KAP1 plays an enhancer role for P-TEFb in HIV-1 proviral transcription even using similar models of latency (McNamara et al., 2016; Morton et al., 2019). While the concept of KAP1 SUMOylation of CDK9 is interesting, many open questions remain from their data. First, their KAP1 RNAi approach elicited induction of HIV-1 promoter activity in a low number of cells, but slightly compromised latent HIV-1 reactivation by the two tested LRAs, potentially consistent with the proposal by McNamara et al. and Morton et al. regarding KAP1’s HIV-1 activating role. A question that remains is why only a small subset of cells were spontaneously, or perhaps stochastically, reactivated in shKAP1 cells and where does the source of heterogeneity come from? In addition, latent HIV-1 reactivation in shKAP1 cells was additive with SAHA treatment in aviremic patient samples, a result that conflicts with their data in the HeLa TZM-bl system in which KAP1 loss compromised SAHA-mediated latent HIV-1 reactivation, an inconsistency that needs further clarification. Finally, SUMO proteins are produced as immature precursors, and require the exposure of a C-terminal GG motif operated by specific proteases to be conjugated. To bypass this step, and increase conjugation efficiency, Ma et al. used a SUMO4 variant mimicking a mature form. Further, unlike SUMO1-3 paralogues, SUMO4 expression is tissue-restricted (Geiss-Friedlander and Melchior, 2007); and thus, definitive evidence that SUMO4 is expressed in immune cells and that its KAP1-mediated conjugation to CDK9 leads to inhibition of HIV-1 transcription awaits validation.

### APOBEC3A Recruitment of KAP1 to 5’-LTR for HIV-1 Transcriptional Repression

Studying the restriction factor APOBEC3A (Apolipoprotein B mRNA editing enzyme catalytic subunit 3A), Taura et al. published in 2019 that APOBEC3A silences HIV-1 gene expression in HeLa and CD4^+^ T cells models of latency and proposed APOBEC3A maintains HIV-1 latency through recruitment of epigenetic silencing machinery, including KAP1, to the 5’-LTR (Taura et al., 2019). This conclusion derived from the following observations. First, CRISPR-Cas9–mediated APOBEC3A knockout in J-Lat 10.6 cells spontaneously induced HIV-1 gene expression (Tat-Rev transcripts) and p24 production while PMA induced HIV-1 gene expression (Tat-Rev transcripts) and p24 production in APOBEC3A knockout cells relative to control cells in a dose-dependent manner, supporting the notion that APOBEC3A silences HIV-1 gene expression. Second, APOBEC3A over-expression in HEK293T cells dampened expression of an HIV-1 luciferase reporter, APOBEC3A specifically bound to the 5’-LTR relative to other genomic regions in J-Lat 10.6 cells, APOBEC3A bound to the 5’-LTR in HeLa TZM-bl cells, and nuclear lysates from HEK293T cells expressing APOBEC3A showed DNA-binding activity with specificity towards the NF-κB/Sp1 sites in the 5’-LTR. Third, transfected APOBEC3A interacted with KAP1 in HEK293T cells, endogenous APOBEC3A bound KAP1 in J-Lat 10.6 cells, and transfected APOBEC3A increased the levels of KAP1, HP1α, and H3K9me3 at the 5’-LTR of the HeLa TZM-bl cell model. Additionally, occupancy of APOBEC3A, KAP1, and H3K9me3 bound to the 5’-LTR in the J-Lat 10.6 cell model significantly decreased upon APOBEC3A knockout relative to control knockout. Fourth, APOBEC3A knockout in primary CD4^+^ T cells increased luciferase levels from infections with both NL4.3/luciferase single-round and replication-competent viruses in the presence of CD3/CD28 stimulation. Fifth, KAP1 knockout in J-Lat 10.6 cells slightly induced HIV-1 activation in the absence of any stimulation (from 1.45 to 2% GFP-positive cells) and in the presence of PMA (from 7.36% to 16.6% GFP-positive cells with 1 nM PMA and from 61.6% to 73.2% GFP-positive cells with 10 nM PMA, respectively), concluding that KAP1 knockout induced spontaneous and enhanced PMA-induced HIV-1 reactivation thus arguing that KAP1 plays a repressive role in HIV-1 transcription. Taken together, Taura et al. proposed a model whereby APOBEC3A binds the 5’-LTR and recruits KAP1 and epigenetic silencing machinery to repress HIV-1 gene expression thereby maintaining latency. Importantly, their studies have limitations that open the door for further research: (1) in all ChIP assays, factor enrichment controls amplifying other genomic (HIV-1 and/or host) sites for specificity were missing and the relative amounts of all factors was unexpectedly high (~2-8% of input DNA), values that are typically not achieved for transcriptional regulators, and (2) KAP1 knockout in primary immune cell models showing KAP1 has repressive effects were not included as it had been done for APOBEC3A. Overall, Taura et al. proposed a novel scaffold for KAP1 recruitment to the HIV-1 5’-LTR to repress transcription (**Figure 4A**), contrasting the canonical ZNF-dependent mechanism (**Figure 1**).

### KAP1-Mediated Degradation of Tat in Myeloid Cells and HIV-1 Transcriptional Repression

In 2021, Ait-Ammar et al. reported that KAP1 represses HIV-1 gene expression in myeloid cells (Ait-Ammar et al., 2021). It was first described that KAP1 overexpression in microglial cells dampened luciferase levels from NL4.3 envelope minus luciferase reporter and that shRNA-mediated KAP1 silencing increased luciferase levels. In correlation with these data, KAP1 occupied the 5’-LTR of a latent GFP-tagged HIV-1 reporter in basal conditions but co-treatment with TNF-α and Hexamethylene bisacetamide (HMBA) reduced KAP1 levels (~2-fold) and induced Pol II occupancy changes, thereby activating the reporter. These studies were then extended to the monocytic THP89 cell model of latency in which shRNA-mediated KAP1 silencing increased (~9-fold) the percentage of GFP positive cells and viral transcripts (Tat and Gag). They then showed KAP1 over-expression in microglial cells decreased Tat activation of an HIV-1 LTR reporter while shRNA-mediated KAP1 silencing facilitated Tat activity. The authors then switched to HEK293T cells to demonstrate that transfected Tat and KAP1 interacted and that increasing amounts of KAP1 over-expression reduced overall Tat levels post-translationally. Finally, they showed that while ectopically expressed KAP1 into HEK293T cells decreased Tat expression, proteasome inhibition with MG132 slightly prevented the Tat reduction, suggesting that protein stability partially explains the mechanism for Tat differences upon KAP1 over-expression. Taken together, using HIV-1 reporter assays with overexpressed or silenced KAP1, Ait-Ammar et al. concluded that KAP1 represses HIV-1 gene expression in myeloid cells and that this is potentially attributed to KAP1-mediated degradation of Tat. Broadly, their studies agree with Ma et al. and Taura et al. in that KAP1 has a repressive role in HIV-1 transcription; however, the three studies provide three different mechanisms for how KAP1 either maintains latency or suppresses the virus upon latency reactivation. Additionally, their results that KAP1 regulates Tat ubiquitination sharply contradict the Morton et al. study that showed KAP1 activates HIV-1 during the host phase in a Tat-independent manner. To mitigate these differences both in mechanism and KAP1 function overall, it will be required to extend the observations by Ait-Ammar et al. to physiologically relevant systems (T cell and primary models with integrated viruses instead of transient reporter assays with KAP1 over-expression) to validate the proposed model that KAP1 is indeed a repressor of HIV-1 through Tat degradation.

### Ectopic Expression of KRAB-ZNF Family Members and HIV-1 Transcriptional Repression

Since KAP1 cooperates with KRAB-ZNF proteins to repress ERVs and the Murine Leukemia Virus (MLV) (Friedman et al., 1996; Wolf and Gofff, 2009; Rowe et al., 2010; Rowe et al., 2013a), in 2012, Nishitsuji et al. found that one member of this family (ZBRK1) negatively regulated HIV-1 LTR driven transcription (Nishitsuji et al., 2012). They observed that ectopic expression of ZBRK1 into HEK293T cells decreased HIV-1 reporter activity without stimulation, diminished synthesis of viral products (p24) from transfected NL4.3 and that shRNA-mediated ZBRK1 silencing conversely induced reporter activity in HEK293T cells and synthesis of viral products (p24) upon infection of MT-4 cells with NL4.3 virus. Given ZNF proteins bind DNA, they then investigated how a potential ZBRK1 DNA-binding function inhibits HIV-1 reporter activity. ZBRK1 inhibited HIV-1 LTR reporters with the following arrangements (-335 to +282 and -245 to +282) but was unable to inhibit reporters with shorter sequences (-106 to +282). Like other KRAB-ZNF family members, ZBRK1 appeared to bind DNA as HEK293T lysates expressing ZBRK1 were able to bind an HIV-1 LTR probe encompassing nucleotides -174 to -95 relative to the site of transcription initiation and transfected ZBRK1 was shown to occupy the 5’-LTR, but negative controls were lacking in this ChIP-qPCR assay. To determine which factors may operate with ZBRK1 to silence HIV-1 LTR under over-expression conditions, they silenced the expression of 3 factors (HP1γ, SETDB1, and KAP1) with siRNA and found that KAP1 silencing (but not SETDB1 and HP1γ) prevented ZBRK1 mediated reporter silencing. Given the collected data, Nishitsuji et al. proposed a model whereby ZBRK1 recruits KAP1 to the HIV-1 LTR reporter to block transcription.

With a similar rationale, in 2015, Nishitsuji et al. (Nishitsuji et al., 2015) screened a targeted library of 52 KRAB-ZNF family members (normally expressed in the pro-monocytic U1 cell line) and identified 5 members (ZNF10, ZNF324, ZNF566, ZNF561, and ZNF333), in addition to ZBRK1 (Nishitsuji et al., 2012), that when over-expressed in HEK293T cells, suppressed HIV-1 LTR reporter activity by more than 50%. Complementary to their over-expression data, siRNA-mediated KRAB-ZNF candidate silencing increased HIV-1 LTR reporter activity in HEK293T cells and shRNA mediated silencing in MT-4 cells augmented the production of viral products (p24) in NL4.3 viral infection assays. Like in their 2012 study (Nishitsuji et al., 2012), using reporter assays in HEK293T cells with plasmids bearing deletions of LTR sequences they provided evidence ZNF10 ectopic expression reduces LTR activity of reporters containing NF-κB and SP1 elements, thus revealing a distinct mode of action compared with ZBRK1, which bound upstream to the NF-kB and Sp1 sites (nucleotides -174 to -95 relative to the site of transcription initiation). Finally, ZNF10 repression activity on reporter assays in HEK293T was compromised when KAP1, HP1γ and SETDB1 expression was silenced (another difference to ZBRK1, which was only compromised with KAP1 co-expression). Taken together the collected data in 2012 and 2015, Nishitsuji et al. concluded that ZBRK1 and ZNF10 require KAP1 to maintain their repressive function. Importantly, in both papers, the authors did not silence nor overexpress KAP1 to determine how KAP1 perturbation alone affected HIV-1 gene expression. Finally, while some of these studies have validated central findings in T cell models (e.g., MT-4 T cells), whether KAP1 cooperates with ZNF proteins to repress HIV-1 in primary models of latency has yet to be formally tested.

### ZNF304-Mediated KAP1 Recruitment to 5’-LTR for HIV-1 Transcriptional Silencing

In 2020 Krasnopolsky et al. (Krasnopolsky et al., 2020) performed a genome-wide CRISPR-Cas9 screen in a Jurkat CD4^+^ T cell line and identified ZNF304 as a silencer of HIV-1 host phase transcription that also dampens the viral phase. Notably, they found that ZNF304 expression was induced by TNF-α (1-10 days post-treatment) in J-Lat 2D10 cells (Pearson et al., 2008) and ZNF304 was detected at the 5’-LTR at baseline and levels increased with TNF-α. Following up on these discoveries, and the fact that ZNF proteins interact with epigenetic silencing machinery including KAP1 and SETDB1, they then examined KAP1 occupancy at the HIV-1 promoter in ZNF304 knockout and control J-Lat 2D10 cells and found reduced KAP1 occupancy upon ZNF304 knockout relative to control knockout. They also observed a reduction in SETDB1 and H3K9me3 occupancy in the same region and provided correlative evidence that loss of ZNF304 diminished the levels of two other repressive epigenetic marks (H3K27me3 and H2K119ub) at the 5’-LTR relative to control cells. They also showed that over-expressed KAP1 interacts with ZNF304 and EZH2, a component of Polycomb Repressive Complexes (PRC2) known to deposit H3K27me3, and that ZNF304 knockout lead to increased Pol II occupancy at the 5’-LTR. Finally, studies in primary CD4^+^ T cells confirmed ZNF304’s repressive role; however, KAP1s repressive function, along with SETDB1 and PRC2, have yet to be established. A model that summarizes the above KAP1–HIV-1 repression studies is presented in **Figure 4A**.

### Current Conundrums of KAP1 Regulation of HIV-1 Gene Expression

We have now summarized the literature pertaining to KAP1 regulation of HIV-1 gene expression thereby opening a conundrum given the conflictive evidence. While McNamara et al. and Morton et al. demonstrated KAP1 knockdown in J-Lat models of latency elicited minimal changes in basal, and a decrease in TNF-α–mediated, HIV-1 transcriptional activation, Ma et al. and Taura et al. proposed KAP1 to be a repressor. However, the results of these two studies did not perfectly align. First, Taura et al. found a minimal spontaneous increase (~1.4-fold) in GFP-positive cells in the J-Lat 10.6 system upon KAP1 knockout and a slight increase upon stimulation (~1.2-2–fold depending on PMA concentration). Second, Ma et al. observed a modest increase (~6-fold) in J-Lat 10.6 GFP-positive cells in both basal, and SAHA and JQ1-stimulated conditions (~1.5-1.9–fold depending on the stimuli used).

Given these observations, the main question becomes, why different labs doing the same experiment with similar, if not identical, latency models and experimental approaches (RNAi and CRISPR-Cas9) obtained different results? Can we call KAP1 a repressor if in only a tiny fraction of cells harboring latent HIV-1 the virus gets reactivated? Is the process of latent HIV-1 reactivation upon KAP1 silencing or depletion in a small number of cells stochastic? Since lentiviruses used to silence (RNAi) and deplete (CRISPR-Cas9) KAP1 expression must be integrated into the Jurkat genome, is it possible that this event elicits unwanted local gene expression changes that trigger latent HIV-1 reactivation? Additionally, if KAP1 participates in the repression of ERVs and other repetitive elements, chronic KAP1 loss would trigger their de-repression which is known to promote activation of neighboring genes and elicit a cell-intrinsic innate immune/inflammatory response potentially contributing to HIV-1 reactivation from latency.

Given what has been done thus far and the large discrepancies among previous studies, it is important that we consider: 1) the models of latency to study KAP1 functions and 2) the experimental approaches that have been used to study KAP1 functions as each approach has its own limitations and caveats. First, the models of latency used to interrogate KAP1’s roles in HIV-1 gene expression regulation are very distinct regarding the cell types used (HeLa TZM-bl, HEK293T, Jurkat T cells) whether they are physiologically relevant or not, whether HIV-1 transcription activity derives from episomal reporters or integrated proviruses. If integrated, one also should consider the HIV-1 integration sites, their chromatin environments, and degrees of responsiveness as the heterogeneity of viral integration (Schroder et al., 2002) may generate proviral quasi-species with potentially diverse modes of transcriptional regulation. Given the selection of HIV-1 proviruses integrated into ZNF genes and repetitive elements of chromosome 19 (Lukic et al., 2014; Jiang et al., 2020), and the known mode of KAP1 repression of these genic and non-genic elements, it sounds appealing to explore whether KAP1 indeed represses this unique class of proviruses. Second, we also need to consider the experimental approaches used to assess KAP1s function. KAP1 silencing through siRNA and shRNA (and even protein overexpression) may elicit unwanted off target effects. Some siRNA/shRNA molecules may target other mRNAs making it impossible to, without a complementation assay, convincingly rule out off target from on target effects. The RNAi approach is currently considered a chronic factor elimination approach because of the time it takes from the moment the mRNA is being silenced to prevent its translation until the moment phenotypes are recorded. Given the issues with RNAi, novel technologies enabling acute and selective control of KAP1 abundance with chemical genetics approaches, such as the “dTAG chemical biology system”, which leverages the potency of cell-permeable heterobifunctional degraders (dTAG-13) (Nabet et al., 2018), can be implemented. This fast chemical genetic tool allows recording of primary phenotypes to delineate causality in gene control, before confounding secondary effects (such as those in chronic RNAi) manifest (Nabet et al., 2018; Jaeger and Winter, 2021). Another tool to assess protein function is ectopic factor expression. While this system has many advantages including time to record phenotypes, factor over-expression typically leads to indirect effects including squelching of regulatory components from other protein complexes and abnormal binding to genomic sites not bound by endogenous proteins. As such, its excessive use without cross-validation with orthogonal approaches makes it difficult to interpret results to confidently conclude the mechanism operates in physiologically relevant systems. Thus, not only more rigorous experimental approaches are needed to settle on this healthy controversy, but also more careful data interpretations. Overall, these ideas raise the question as to which systems and experimental approaches are best, or at the very least, a better option, to study KAP1 and HIV-1 that will settle this controversy.

## KAP1 Gene Expression Regulation of Other Viruses

In addition to regulating ERVs and HIV-1, KAP1 also controls other viruses. This section will discuss how KAP1 either maintains latency, promotes reactivation from latency, and/or regulates viral replication in DNA and RNA viruses.

### RNA Viruses

One of the first viruses to be characterized as KAP1 regulated was the MLV retrovirus. Upon reverse transcription and integration into the host genome, MLV is transcriptionally silenced by protein complexes that bind the Primer Binding Site (PBS) at the 5’-end of the MLV genome (Wolf and Goff, 2007). Specifically, pioneer work by the Goff lab has shown that KAP1 occupies the PBS sequence and that silencing of KAP1 using siRNA relieved the repression of MLV in embryonic carcinoma cells (F9 cells), suggesting that KAP1 is required for MLV latency maintenance. Further investigation showed that the KAP1 HP1-binding motif (PxVxL) (**Figure 2A**) was required for MLV repression (Wolf et al., 2008a), and that ZFP809, a KRAB-domain containing ZFP recruits KAP1 to the PBS (Wolf and Goff 2009), indicating that the canonical mode of KAP1-mediated repression of ERVs (**Figure 1**) may be utilized for KAP1 repression of MLV. Additionally, another study from the Goff lab found that KAP1 SUMOylation, particularly at residue K779, is required for KAP1-dependent repression of MLV (Lee et al., 2018), consistent with previous reports linking KAP1 SUMOylation and transcriptional repression in reporter assays (Mascle et al., 2007). Interestingly, recent studies demonstrate KAP1 inhibits replication of another retrovirus, Prototype Foamy Virus (PFV) (Yuan et al., 2021). While Yuan et al. showed that KAP1 maintains, or its expression is required to maintain, repressive histone marks at the PFV LTR promoter (consistent with the KAP1-ERV repression model), the authors also discovered that KAP1 overexpression decreases protein levels of the PFV viral transactivator Tas in a proteasome-dependent manner, suggesting that KAP1 marks Tas for degradation to restrict PFV (Yuan et al., 2021), like the KAP1-Tat degradation signature Ait-Ammar found in microglial cells (Ait-Ammar et al., 2021).

Besides retroviruses, KAP1 regulates other RNA viruses including influenza. In 2019, Schmidt et al. discovered that KAP1 SUMOylation was decreased upon infection of cell models with Influenza A Virus (Schmidt et al., 2019). Using reconstitution assays in A549 lung cancer cells upon IAV infection, the authors showed that KAP1 knockout using CRISPR-Cas9 led to decreased viral replication, and that KAP1, but not KAP1 mutated at 6 Lysine residues capable of getting SUMOylated (named SUMO mutant), could rescue replication levels upon KAP1 loss, suggesting that SUMOylation is critical for productive IAV infection. Using transcriptome profiling, the authors expressed wild-type KAP1 and the SUMO mutant in KAP1 knockout cells to show that SUMOylation is important for KAP1-mediated repression of ERVs and antiviral gene products, including genes related to innate immunity. Notably, another group studying highly pathogenic IAV found that KAP1 phosphorylation at S473 (pS473) (**Figure 2A**), not SUMOylation, was involved in regulating immune responses upon IAV infection (Krischuns et al., 2018). Using KAP1 knockout cell models in response to IAV infection, they found that loss of KAP1 increased expression of inflammatory genes (e.g., IL-6) and decreased viral (VSV-luc) infection rate, suggesting KAP1 is a negative regulator of innate immunity during IAV infection. Interestingly, reconstitution assays showed that KAP1 pS473 phosphomimetic elicited upregulated transcription of antiviral gene signatures and decreased viral replication rates, suggesting that site-specific KAP1 phosphorylation promotes KAP1-mediated innate immunity triggering (Krischuns et al., 2018).

KAP1 has also been implicated in SARS-CoV-2 regulation. In 2021, Tovo et al. conducted a clinical correlation study showing that children infected with SARS-CoV-2 had upregulated levels of KAP1 and SETDB1 along with type 1 interferon-stimulated genes, proposing that KAP1 may play a repressive role in infection (Tovo et al., 2021). In addition to this correlational study, another group found that KAP1 silencing using siRNA led to enhanced expression of ACE2, the SARS-CoV-2 receptor, in cancer cell models and primary human lung epithelial cells (Wang et al., 2021). Despite these two studies, the mechanism of action and whether KAP1 truly plays a repressive role in the SARS-CoV-2 infection remains unclear.

### DNA Viruses

Herpesviruses such as Epstein Barr virus (EBV), Kaposi’s Sarcoma-associated Herpesvirus (KSHV), and human cytomegalovirus (HCMV) were all shown to be regulated by KAP1. This section will highlight the status of the literature pertaining to KAP1 regulation of this class of viruses.

Reports have established that KAP1 maintains both EBV and KSHV latency by binding to the promoters of viral genes to inhibit their expression by regulating occupancy of the repressive machinery (e.g., HP1) (Chang et al., 2009; Bentz et al., 2015), potentially mirroring the mechanism of ERV repression (**Figure 1**). Notably, in both EBV and KSHV, KAP1 repressive role can be countered when phosphorylated at S824 (pS824) within the C-terminal region (**Figure 2A**), signifying that this post-translational modification (PTM) can prevent KAP1-dependent repression (Bentz et al., 2015; Li et al., 2019). Recent reports have also demonstrated that when patients infected with *Plasmodium falciparum* and have secondary infection with EBV receive chloroquine, an agonist of the Ataxia Telangiectasia Mutated kinase and a drug that the parasite is susceptible to, EBV escapes latency and begins to replicate, which coincides with KAP1 pS824 and DNA repair-independent activation of EBV (Li et al., 2017b).

In the context of HCMV, Rauwel et al. showed that KAP1 phosphorylation (pS824) acts as a “switch” for HCMV activation (Rauwel et al., 2015). Here, shRNA-mediated silencing of KAP1 in cord blood CD34^+^ cells increased HCMV expression of early and late HCMV genes compared to control cells, suggesting that KAP1 is required for HCMV latency maintenance. The authors then showed that upon HCMV activation with dendritic cell differentiation, KAP1 continued to occupy the viral genome while SETDB1 and H3K9me3 occupancy at the early and latent gene promoters decreased as expected given latency escape. The authors demonstrated that KAP1 S824 was phosphorylated by mTOR upon differentiation and HCMV activation, and that KAP1 pS824 remained on chromatin and potentially regulated HCMV activation. Supporting this notion, pharmacological induction of KAP1 pS824 using chloroquine led to HCMV latency reversal, indicating that KAP1 site-specific phosphorylation acts as a switch to relieve KAP1 maintenance of HCMV latency.

Overall, a common theme is site-specific KAP1 PTMs (e.g., pS473, pS824 and SUMOylation) (**Figure 2A**), which act as possible switches between latency and reactivation for a diverse set of viruses. However, it remains unclear whether KAP1 phosphorylation only blocks KAP1 repressive functions or if phosphorylation converts KAP1 into a transcriptional activator. Mechanistic interrogation using precise tools in physiological models will be required to define how KAP1 PTMs exert their transcriptional functions and whether they are required for, or a consequence of, transcriptional activation to provide causality in KAP1 gene expression control.

## KAP1 Regulation of Cell Fate Responses and Immune Cell Functions

In addition to regulating a diverse set of viruses, KAP1 has been implicated in a variety of host biological processes including innate immunity, tumor microenvironment regulation, and immune cell development, among others (Czerwinska et al., 2020; Lee et al., 2020; Liang et al., 2020; Liu et al., 2020; Chikuma et al., 2021; Park et al., 2021) (**Figure 3**). While some of these reported KAP1-dependent functions are mediated by KAP1 repression of ERVs (Tie et al., 2018; Schmidt et al., 2019; Lee et al., 2020), many of these cellular roles have been mediated by KAP1 regulating transcription of host genes (Kamitani et al., 2008; Wang et al., 2017) or through KAP1 utilizing its E3 ubiquitin ligase function to degrade specific factors (Liang et al., 2011; Qin et al., 2021).

To assess the phenotypic consequences of immune cell differentiation and function, many groups have conditionally removed KAP1 from mouse models and observed a plethora of developmental defects. In 2012, Chikuma et al. reported that T cell specific knockout of KAP1 led to an increase of autoreactive CD4^+^ T helper 17 (Th17) cells, decreases in IL-2 production, and early death due to spontaneous autoimmunity (Chikuma et al., 2012). Notably, even though IL-2 regulates Foxp3^+^ T regulatory (Tregs) cells, Tregs were also accumulated in their KAP1 knockout model likely due to upregulation of TGF-β cytokines. Notably, Tanaka et al. has also seen similar effects with regards to Treg development in that Treg-specific knockout of KAP1 led to spontaneous autoimmune diseases (e.g., lymphadenopathy, lung inflammation, and an increased number of immune cells in the colonic lamina propria) (Tanaka et al., 2018). They also showed that KAP1 loss impaired the proliferation and capacity of Tregs to suppress effector T cells growth. Mechanistically, they showed that a large subset of Tregs signature genes were dysregulated in KAP1-deficient Tregs including Foxp3 and metabolism related-target genes (e.g., Slc1a5 and mTORC1 activated) (Tanaka et al., 2018). While other groups have reported similar findings in Th17 populations (Jiang et al., 2018; Gehrmann et al., 2019), Santoni de Sio et al. stated that B lymphoid-specific KAP1 knockout reduced the number of mature B cells thereby suggesting that KAP1 controls B cell development in addition to T cell development (Santoni de Sio et al., 2012).

Given that KAP1 regulates viruses in both basal and stimulated conditions (as described in earlier sections) and that viral infection induces immune responses, it is not surprising that KAP1 disruption also affects immune cell functions in virus-independent and -dependent manners. Because KAP1 regulates transcription of both ERVs and target genes in human cells that regulate differentiation, it is also not surprising that T and B cell differentiation into immune effector subtypes would be affected upon KAP1 loss. However, it remains unclear mechanistically why KAP1 loss affects specific subtypes in the T cell differentiation process. What transcriptional mechanisms is KAP1 exerting to precisely regulate specific T cell functions in Th17 and Tregs cell populations? Additionally, does KAP1 have universal or cell-type specific functions? Related to this question, are some cell-types (e.g., Tregs and Th17) more susceptible to perturbations (e.g., ERV disruption)? Because Th17 and Tregs have opposing immune regulatory functions, one would expect that KAP1 control of gene regulatory networks (both activation and repression) evolved lineage-committed programs. Given the importance of KAP1 interaction with sequence- and pathway-specific transcription factors (Bacon et al., 2020), cell-type specific KAP1 action may be dictated by KAP1’s interaction with one or more cell-type, sequence-specific regulatory factors like RORγT in Th17 and Foxp3 in Tregs, as reported by Tanaka et al. (Tanaka et al., 2018).

Finally, the pleiotropic effects observed in the studies outlined in this review require careful molecular dissection to answer these questions. Understanding how KAP1 directly affects transcription (or non-transcription functions) in immune models will help delineate causality in KAP1 regulation of the various phenotypes and inform about the best therapeutic strategies to cope with the diseased states.

## Concluding Remarks

After this journey we have learned that KAP1 regulates transcriptional repression and activation thereby contributing to multiple important phenotypes from the control of embryo development to the regulation of stem cell maintenance and differentiation repression. Given these essential functions, KAP1 inactivation triggers disease phenotypes such as embryonic lethality, cancer, and obesity. Besides these broad cellular functions and phenotypes, KAP1 has also been linked to the control of viral expression (including HIV-1 and a large diversity of DNA and RNA viruses) either positively or negatively. For HIV-1, our current understanding of KAP1 control of latency and reactivation needs further research to distinguish direct versus indirect consequences of KAP1 loss on proviral expression. These studies will provide causality in HIV-1 gene expression regulation, define whether KAP1 is a positive or negative regulator, and whether KAP1 operates at the transcriptional or other levels (e.g., post-transcriptional and/or post-translational). Notably, KAP1 was first described to restrict HIV-1 by interacting with Integrase to block viral integration into host chromatin (Allouch et al., 2011). This discovery offers the possibility that KAP1 can functionally link the processes of integration and transcription and provides the rationale for investigating non-transcription KAP1 functions in the gene expression cycle.

Since KAP1 has been proposed to both repress (**Figure 4A**) and activate (**Figure 4B**) HIV-1 transcription, we posit the paradigm that KAP1 regulates a switch from repression to activation (referred to as “repression-activation switch” (**Figure 4C**)). In this regulatory switch, viruses and immune programs may utilize a transcriptional repressor for their own activation by promoting KAP1 site-specific phosphorylation (pS473 and/or pS824) thereby enhancing viral gene expression for infection and immune gene expression for accurate cell fate responses. While it remains unknown if HIV-1 utilizes this “switch” mechanism, HCMV may utilize this strategy to disrupt KAP1-repression functions and convert KAP1 into an activator. Notably, in 2015 the Trono lab proposed a KAP1 pS473 requirement for myoblast differentiation where KAP1 represses muscle-specific genes in undifferentiated myoblasts but then is required for their activation upon differentiation of myoblasts to myotubes (Singh et al., 2015). KAP1 S473 phosphorylation disrupted KAP1 interaction with transcriptional repressive machinery, thereby enabling MyoD-dependent transcriptional activation of target gene expression leading to efficient myoblast differentiation. This discovery indeed suggests that a KAP1 repression-activation switch may operate in other contexts and is worth investigating.

**Figure 5:**
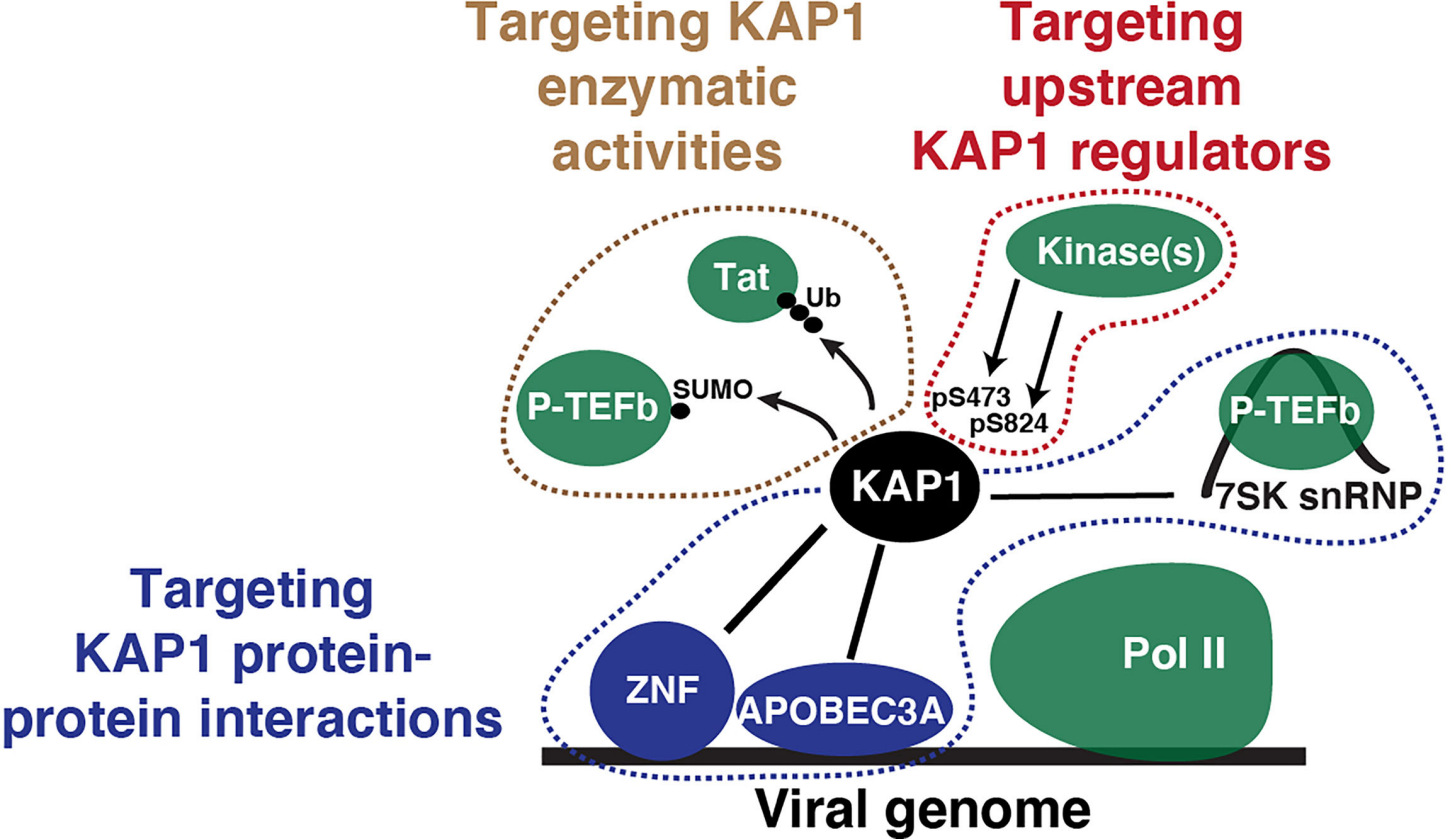
Potential therapeutic targeting strategies to interrupt KAP1 functions in the context of viral infection. Simple scheme depicting KAP1 functions (enzymatic activities, protein-protein interactions, and upstream regulators) that can be targeted to derail viral infection and other diseases states.

While further work is required to determine if KAP1 operates as a transcriptional switch, the models described in this review by several groups has opened potential therapeutic opportunities to target KAP1 during viral infection or latency (**Figure 5**). First, KAP1 has enzymatic activities (SUMO and Ubiquitin ligase), which could be interrupted to prevent KAP1 from targeting P-TEFb/Tat (as Ma et al. and Ait-Ammar et al. proposed) to potentially promote latency reactivation. Second, if KAP1 interacts with other factors to regulate HIV-1 transcription (such as P-TEFb as described in McNamara et al. and Morton et al. or APOBEC3A as described in Taura et al.) blocking these protein-protein interactions with specific small molecules could block KAP1-dependent functions (transcriptional activation or repression, respectively). Importantly, this daunting task will require both structural studies to precisely identify the KAP1-protein interaction pockets and large-scale screens to identify lead compounds that inhibit these interactions. Third, upstream regulators such as DNA-PK could also be inhibited using commercially available small molecules (e.g., NU7441), which could prevent KAP1 phosphorylation and thus latent HIV-1 reactivation; however, the off-target effects for targeting critical kinases will have to be carefully contemplated. While these ideas present intriguing therapeutic opportunities, dissecting the exact molecular mechanisms underlying KAP1 regulation of HIV-1 will be required to make the much-needed progress towards achieving the long-term therapeutic goal.

Given the broad utilization of repressive and activating modes of gene regulation, is KAP1 the “jack-of-all-trades” or are the phenotypes observed a consequence of the loss of KAP1 functions due to its chronic silencing or depletion? Are the *in vitro* cellular phenotypes a direct consequence of the loss of KAP1 repressive or activating functions, or are they a result of KAP1 long-term loss? Because KAP1 represses the expression of transposable elements, whose de-repression is known to activate neighboring genes, does KAP1 loss provoke the indirect activation of non-target genes through this mechanism or others? Many questions remain unanswered, and studies are needed to truly describe how site-specific phosphorylation and/or SUMOylation tune KAP1 transcription functions and interactions with proteins required for gene repression or activation. Which phosphorylation mark and/or SUMOylation residue is the most critical for viral activation and/or repression? Do these modifications affect KAP1 enzymatic activity, or do they solely affect KAP1s scaffolding roles? It will be interesting to see how the field refines the current dogmas.

Collectively, future studies must leverage chemical genetics approaches for acute KAP1 elimination to rule out cumbersome indirect effects and thus provide direct evidence that KAP1 functions as a repressor, activator, or repressor-activator switch of the system under interrogation. Addressing these questions will surely bolster our understanding of the precise and specific mechanisms of KAP1 regulation of viral and cellular programs, how KAP1 maintains key functions to prevent disease, and what best strategies can be deployed to cope with KAP1 inactivation in diseases states.

## Author Contributions

All authors contributed equally. All authors contributed to the article and approved the submitted version.

## Funding

This study was supported by the National Institute of Allergy and Infectious Diseases (NIAID) of the NIH under award numbers R01AI114362 (to ID’O) and 5T32AI007530 (to KR), and the National Cancer Institute of the NIH under award numbers 1R03CA259672 (to ID’O) and F99CA264296 (to UH).

## Conflict of Interest

The authors declare that the research was conducted in the absence of any commercial or financial relationships that could be construed as a potential conflict of interest.

## Publisher’s Note

All claims expressed in this article are solely those of the authors and do not necessarily represent those of their affiliated organizations, or those of the publisher, the editors and the reviewers. Any product that may be evaluated in this article, or claim that may be made by its manufacturer, is not guaranteed or endorsed by the publisher.
